# Supplementary material for: A novel splice variant of Elp3/Kat9 regulates mitochondrial tRNA modification and function
Source: Sci Rep. 2022 Aug 31;12:14804. doi: 10.1038/s41598-022-18114-x (PMC9433433; doi:10.1038/s41598-022-18114-x)
Supplement: Supplementary file 1 — Supplementary Information. [file 41598_2022_18114_MOESM1_ESM.pdf]

Supplementary Information

A novel splice variant of Elp3/Kat9 regulates mitochondrial tRNA modification and function

Rachid Boutoual[1*], Hyunsun Jo[2], Indra Heckenbach[1,3], Ritesh Tiwari[1], Herbert Kasler[1], Chad A. Lerner[1], Samah Shah[1], Birgit Schilling[1], Vincenzo Calvanese[2], Matthew J. Rardin[4], Morten Scheibye-Knudsen[3], and Eric Verdin[1,2*].

Affiliations:

[1] Buck Institute for Research on Aging, 8001 Redwood Boulevard, Novato, CA 94945, USA.
[2] Gladstone Institutes and University of California, San Francisco, San Francisco, CA 94158, USA.
[3]Center for Healthy Aging, Department of Cellular and Molecular Medicine, University of Copenhagen, Copenhagen, Denmark [4]Amgen Research, Amgen Inc., South San Francisco, CA 94080, USA.

*Correspondence: RBoutoual@buckinstitute.org, EVerdin@buckinstitute.org.

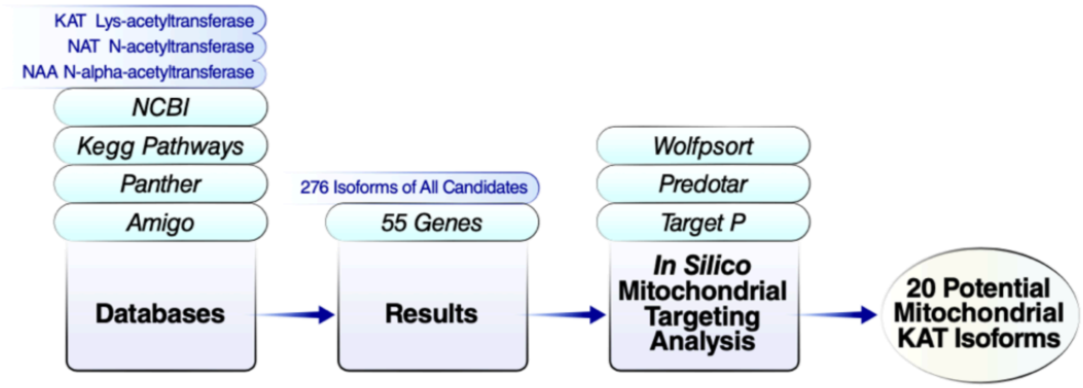

Figure S1. The methodology used to identify putative mitochondrial acetyltransfereases.

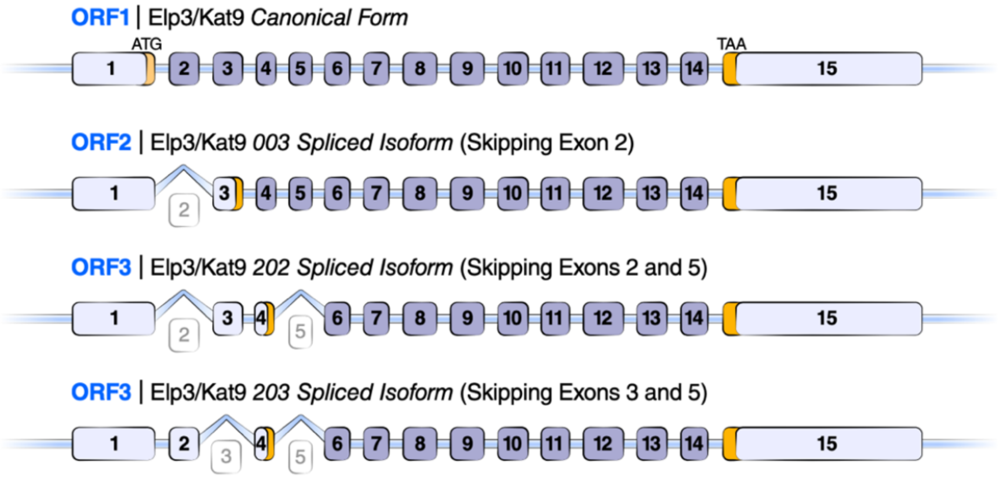

Figure S2. Representation of full-length ELP3 and its spliced isoforms. Start and stop codon are highlighted in each isoform.

Figure S3. Primary sequences of ELP3, mRNAs and proteins

Homo sapiens elongator acetyltransferase complex subunit 3 (ELP3), transcript variant 1, mRNA (canonical ELP3)

<u>mRNA</u>

NCBI Reference Sequence: NM_018091.5
LOCUS NM_018091 3232 bp

GTGCACGTCGGCTTCCGGGAAGAGCTTTACGATACATTGACCGACATTTTACGACAGGCGGGATTGTTTTGTGGCT
GTCAGCTTTCCCCGTGGTCTGAGTTTGTGGCTGCATTTTTATCTCTGGTGGCTCTGCTACGGCGGCGCAGAAATGA
GGCAGAAGCGGAAAGGAGATCTCAGCCCTGCTGAGCTGATGATGCTGACTATAGGAGATGTTATTAAACAACTGA
TTGAAGCCCACGAGCAGGGGAAAGACATCGATCTAAATAAGGTGAAAACCAAGACAGCTGCCAAATATGGCCTTT
CTGCCCAGCCCCGCCTGGTGGATATCATTGCTGCCGTCCCTCCTCAGTATCGCAAGGTCTTGATGCCCAAGTTAAA
GGCGAAACCCATCAGAACTGCTAGTGGGATTGCTGTCGTGGCTGTGATGTGCAAACCCCACAGATGTCCACACAT
CAGTTTTACAGGAAATATATGTGTATACTGCCCTGGTGGACCTGATTCTGATTTTGAGTATTCCACCCAGTCTTACA
CTGGCTATGAGCCAACCTCCATGAGAGCTATCCGTGCCAGATATGACCCTTTCCTACAGACAAGACACCGAATAGA
ACAGTTAAAACAACTTGGTCATAGTGTGGATAAAGTGGAGTTTATTGTGATGGGTGGAACGTTTATGGCCCTTCCA
GAAGAATACAGAGATTATTTTATTCGAAATTTACATGATGCCTTATCAGGACATACTTCCAACAATATTTACGAGGC
AGTCAAGTATTCTGAGAGAAGCCTCACAAAGTGTATTGGAATTACTATTGAAACCAGACCAGATTACTGCATGAAG
CGACATTTAAGTGACATGTTGATTCCAACAATATTTACGAGGCACCTATGGCTGCACAAGGCTGGAGATTGGGGT
GCAGAGTGTTTATGAAGATGTGGCTAGAGACACCAACAGGGGCCACACTGTGAAGGCAGTGTGTGAGTCATTTCA
CCTGGCCAAAGATTCCGGTTTTAAAGTGGTGGCCCATATGATGCCTGACCTGCCAAACGTGGGACTAGAAAGAGA
CATTGAACAGTTCACAGAGTTTTTTGAGAACCCTGCTTTTCGTCCCGATGGGCTGAAACTCTATCCTACCCTGGTGA
TTCGTGGGACCGGGCTTTATGAGCTTTGGAAATCAGGAAGATATAAGAGTTACTCTCCTAGTGACCTGGTTGAATT
GGTGGCTCGGATCCTAGCCCTCGTGCCTCCATGGACTCGAGTGTACCGAGTACAGAGGGATATTCCAATGCCTTTA
GTTAGCTCAGGAGTAGAGCATGGTAACCTGAGAGAGCTGGCACTTGCAAGAATGAAAGACCTCGGAATACAGTG
TCGAGATGTGAGAACCAGAGAAGTTGGAATCCAAGAAATTCATCACAAAGTACGGCCATACCAGGTTGAATTGGT
AAGGAGAGATTATGTTGCAAATGGTGGCTGGGAAACATTCTTGTCATACGAAGACCCAGATCAAGACATTTTGAT
TGGCCTCCTACGATTACGCAAGTGTTCAGAAGAAACTTTCCGTTTCGAATTGGGTGGAGGTGTCTCCATAGTACGA
GAGCTGCATGTGTATGGGAGTGTGGTCCCTGTGAGCAGCCGGGATCCTACTAAATTTCAGCATCAGGGATTTGGC
ATGCTGCTGATGGAGGAAGCAGAAAGAATAGCTAGAGAAGAACATGGGTCTGGGAAAATCGCTGTGATATCAGG
GGTCGGCACCAGGAATTATTATAGAAAGATCGGCTACAGATTACAAGGCCCGTACATGGTGAAGATGCTGAAATA
ATGGCCACACCAGTCCACTCTTCTGCAGTATCCTCCCTGGCAGAACACGGAGAATCAGGATTTCTTAAATACTCAAC
AGAGAGGCTGAGCAGAGCAAATGGGGGGCTTCACCCTCATCCCGCAGCTGCAGAGACTGGAAACTGCCTTCAAG
GCCACGGCTGGTCATCTGCTGACCACACCCCAGATCCGCCCTCTCCTGCGTGCACCCCAAAAAATCACTTGCGTTTT
TGAGGCTTAAATCATCTATCCAGTTTCTACATTTTGCATGAGGCCTGCAGGTGGCCTATTTTGACTCAGACGGTGAA
AAAAGCAAATTAACTCATTTGGACACCATAACTCATGCAATAAAACTGATTGTCATTCGAGGAGCAAACTTAAGAG
TAGTTTATTTATATACCCTGGGGACAGAAAGTCAGGTTGAAACAGGAAAACCACCAGACTCTAATCTCAGCCCTTT
AACGACATACGCATTGGAGCGCAAGTTAGGAAAATGAGCTTTTGTTTTCATGGAAATCATTCTGATTACAGTGCTG
ATGTTTAGAAATAAATAGCAGTGTGACTGGGAAAGAGGAATTGCAGTTGTGGGGGTGTGAGCCTGGCAGCAGCC
AGCCAGCAGCCTCTCCCAGGCGGGAGTCTACCATCCGAGACGGCGATGACAAAGAGCTTCATTCCACATTCTTTGT
TATCTCTACTTCCCACCCTCTTGGCAACTACAGAGCAGTGTGGGCAGCCCCAAGTGTGGTCCCCAGAGAGCGTTTG

GCTTTCCTGTCTGTCTATCCTGAGCGGGTGGAGTCTCAGGTTGTGTGCCCCTAAATCAAGATTTGCTTCCACAGAAG
CCATTACTTGCAATTTTTTTTTTTTTTCTGAGAAAGTCTCGCTGTGTCACCCAGGCTGGAGTGCAGTGGCGCAATCT
CACTGCATCCTCCGCCTCCCGGGTTCAAGCGATTCTCCCGCCTCAGCCTCCTGAGTAGCTGGGATTACAGGCACCC
GCCGCTGCTAATTTTTGTATTTTTAGTAGAGATGGGGGTTTCACCATATTGGTCAGGCTGGTCTCGAACTCCTGACC
TCAGGTGATCAACCCACCTTGGCCTCCCTAAATGCCGGGATTACAGGCATGAGCCACCGCTCCCAGCCTTTGATTTT
TTAAGGTGGATTTTGGTTGTTATAAATGGAGAAAGGTAAGAGTTCAAGTTCAACCCGTGTGTGAAAGCAAAACAA
TGGAAAACAGGATTGGCTTCTTCAAAGGCTCCTCTTGTAGAACTGCCTCTTTGAAATTTCGAGGTAATCTACTTTGG
AGACTCTGCCTGGAGAGGGTCAGTTCCTAAGTTAAAAGCATCGCTTAACCTTGGCTCCTGTGGCATTTTACAAAGG
TTTAAAGGAATTGATTCCTCTGAAAGGGCCTGAAAATAAAAAGTCTTTAACATACAAAAAAAAAAAAAAA

Protein

NCBI Reference Sequence: NP_060561.3
LOCUS NP_060561 547 aa

MRQKRKGDLSPAELMMLTIGDVIKQLIEAHEQGKDIDLNKVKTKTAAKYGLSAQPRLVDIIAAVPPQYRKVL
MPKLKAKPIRTASGIAVVAVMCKPHRCPHISFTGNICVYCPGGPDSDFEYSTQSYTGYEPTSMRAIRARYDPF
LQTRHRIEQLKQLGHSVDKVEFIVMGGTFMALPEEYRDYFIRNLHDALSGHTSNNIYEAVKYSERSLTKCIGITI
ETRPDYCMKRHLSDMLTYGCTRLEIGVQSVYEDVARDTNRGHTVKAVCESFHLAKDSGFKVVAHMMPDLP
NVGLERDIEQFTEFFENPAFRPDGLKLYPTLVIRGTGLYELWKSGRYKSYSPSDLVELVARILALVPPWTRVYRV
QRDIPMPLVSSGVEHGNLRELALARMKDLGIQCRDVRTREVGIQEIHHKVRPYQVELVRRDYVANGGWETF
LSYEDPDQDILIGLLRLRKCSEETFRFELGGGVSIVRELHVYGSVVPVSSRDPTKFQHQGFGMLLMEEAERIAR
EEHGSGKIAVISGVGTRNYYRKIGYRLQGPYMVKMLK

Homo sapiens elongator acetyltransferase complex subunit 3 (ELP3), transcript variant 4, mRNA (Exon3 and Exon5 Skipping)

mRNA

NM_001284224.1
LOCUS NM_001284224 3032 bp

GTATTCTGAGAGAAGCCTCACAAAGTGTATTGGAATTACTATTGAAACCAGACCAGATTACTGCATGAAGCGACAT
TTAAGTGACATGTTGACCTATGGCTGCACAAGGCTGGAGATTGGGGTGCAGAGTGTTTATGAAGATGTGGCTAGA
GACACCAACAGGGGCCACACTGTGAAGGCAGTGTGTGAGTCATTTCACCTGGCCAAAGATTCCGGTTTTAAAGTG
GTGGCCCATATGATGCCTGACCTGCCAAACGTGGGACTAGAAAGAGACATTGAACAGTTCACAGAGTTTTTTGAG
AACCCTGCTTTTCGTCCCGATGGGCTGAAACTCTATCCTACCCTGGTGATTCGTGGGACCGGGCTTTATGAGCTTTG
GAAATCAGGAAGATATAAGAGTTACTCTCCTAGTGACCTGGTTGAATTGGTGGCTCGGATCCTAGCCCTCGTGCCT
CCATGGACTCGAGTGTACCGAGTACAGAGGGATATTCCAATGCCTTTAGTTAGCTCAGGAGTAGAGCATGGTAAC
CTGAGAGAGCTGGCACTTGCAAGAATGAAAGACCTCGGAATACAGTGTCGAGATGTGAGAACCAGAGAAGTTGG
AATCCAAGAAATTCATCACAAAGTACGGCCATACCAGGTTGAATTGGTAAGGAGAGATTATGTTGCAAATGGTGG
CTGGGAAACATTCTTGTCATACGAAGACCCAGATCAAGACATTTTGATTGGCCTCCTACGATTACGCAAGTGTTCA

GAAGAAACTTTCCGTTTCGAATTGGGTGGAGGTGTCTCCATAGTACGAGAGCTGCATGTGTATGGGAGTGTGGTC
CCTGTGAGCAGCCGGGATCCTACTAAATTTCAGCATCAGGGATTTGGCATGCTGCTGATGGAGGAAGCAGAAAGA
ATAGCTAGAGAAGAACATGGGTCTGGGAAAATCGCTGTGATATCAGGGGTCGGCACCAGGAATTATTATAGAAA
GATCGGCTACAGATTACAAGGCCCGTACATGGTGAAGATGCTGAAATAATGGCCACACCAGTCCACTCTTCTGCA
GTATCCTCCCTGGCAGAACACGGAGAATCAGGATTTCTTAAATACTCAACAGAGAGGCTGAGCAGAGCAAATGGG
GGGCTTCACCCTCATCCCGCAGCTGCAGAGACTGGAAACTGCCTTCAAGGCCACGGCTGGTCATCTGCTGACCACA
CCCCAGATCCGCCCTCTCCTGCGTGCACCCCAAAAAATCACTTGCGTTTTTGAGGCTTAAATCATCTATCCAGTTTCT
ACATTTTGCATGAGGCCTGCAGGTGGCCTATTTTGACTCAGACGGTGAAAAAAGCAAATTAACTCATTTGGACACC
ATAACTCATGCAATAAAACTGATTGTCATTCGAGGAGCAAACTTAAGAGTAGTTTATTTATATACCCTGGGGACAG
AAAGTCAGGTTGAAACAGGAAAACCACCAGACTCTAATCTCAGCCCTTTAACGACATACGCATTGGAGCGCAAGTT
AGGAAAATGAGCTTTTGTTTTCATGGAAATCATTCTGATTACAGTGCTGATGTTTAGAAATAAATAGCAGTGTGAC
TGGGAAAGAGGAATTGCAGTTGTGGGGGTGTGAGCCTGGCAGCAGCCAGCCAGCAGCCTCTCCCAGGCGGGAGT
CTACCATCCGAGACGGCGATGACAAAGAGCTTCATTCCACATTCTTTGTTATCTCTACTTCCCACCCTCTTGGCAACT
ACAGAGCAGTGTGGGCAGCCCCAAGTGTGGTCCCCAGAGAGCGTTTGGCTTTCCTGTCTGTCTATCCTGAGCGGG
TGGAGTCTCAGGTTGTGTGCCCCTAAATCAAGATTTGCTTCCACAGAAGCCATTACTTGCAATTTTTTTTTTTTTTCT
GAGAAAGTCTCGCTGTGTCACCCAGGCTGGAGTGCAGTGGCGCAATCTCACTGCATCCTCCGCCTCCCGGGTTCAA
GCGATTCTCCCGCCTCAGCCTCCTGAGTAGCTGGGATTACAGGCACCCGCCGCTGCTAATTTTTGTATTTTTAGTAG
AGATGGGGGTTTCACCATATTGGTCAGGCTGGTCTCGAACTCCTGACCTCAGGTGATCAACCCACCTTGGCCTCCC
TAAATGCCGGGATTACAGGCATGAGCCACCGCTCCCAGCCTTTGATTTTTTAAGGTGGATTTTGGTTGTTATAAATG
GAGAAAGGTAAGAGTTCAAGTTCAACCCGTGTGTGAAAGCAAAACAATGGAAAACAGGATTGGCTTCTTCAAAG
GCTCCTCTTGTAGAACTGCCTCTTTGAAATTTCGAGGTAATCTACTTTGGAGACTCTGCCTGGAGAGGGTCAGTTCC
TAAGTTAAAAGCATCGCTTAACCTTGGCTCCTGTGGCATTTTACAAAGGTTTAAAGGAATTGATTCCTCTGAAAGG
GCCTGAAAATAAAAAGTCTTTAACATACAAAAAAAAAAAAAAAAAA

Protein

NCBI Reference Sequence: NP_001271153.1
LOCUS NP_001271153 428 aa

MSTHQFYRKYMCPTSMRAIRARYDPFLQTRHRIEQLKQLGHSVDKVEFIVMGGTFMALPEEYRDYFIRNLH
DALSGHTSNNIYEAVKYSERSLTKCIGITIETRPDYCMKRHLSDMLTYGCTRLEIGVQSVYEDVARDTNRGHTV
KAVCESFHLAKDSGFKVVAHMMPDLPNVGLERDIEQFTEFFENPAFRPDGLKLYPTLVIRGTGLYELWKSGRY
KSYSPSDLVELVARILALVPPWTRVYRVQRDIPMPLVSSGVEHGNLRELALARMKDLGIQCRDVRTREVGIQE
IHHKVRPYQVELVRRDYVANGGWETFLSYEDPDQDILIGLLRLRKCSEETFRFELGGGVSIVRELHVYGSVVPV
SSRDPTKFQHQGFGMLLMEEAERIAREEHGSGKIAVISGVGTRNYYRKIGYRLQGPYMVKMLK

Homo sapiens elongator acetyltransferase complex subunit 3 (ELP3), transcript variant 5, mRNA (Exon2 and Exon5 skipping)

mRNA

NCBI Reference Sequence: NM_001284225.1
LOCUS NM_001284225 3071 bp

GTGCACGTCGGCTTCCGGGAAGAGCTTTACGATACATTGACCGACATTTTACGACAGGCGGGATTGTTTTGTGGCT
GTCAGCTTTCCCCGTGGTCTGAGTTTGTGGCTGCATTTTTATCTCTGGTGGCTCTGCTACGGCGGCGCAGAAATGA
GGCAGAAGCGGAAAGGGTGAAAACCAAGACAGCTGCCAAATATGGCCTTTCTGCCCAGCCCCGCCTGGTGGATAT
CATTGCTGCCGTCCCTCCTCAGTATCGCAAGGTCTTGATGCCCAAGTTAAAGGCGAAACCCATCAGAACTGCTAGT
GGGATTGCTGTCGTGGCTGTGATGTGCAAACCCCACAGATGTCCACACATCAGTTTTACAGGAAATATATGTGTCC
AACCTCCATGAGAGCTATCCGTGCCAGATATGACCCTTTCCTACAGACAAGACACCGAATAGAACAGTTAAAACAA
CTTGGTCATAGTGTGGATAAAGTGGAGTTTATTGTGATGGGTGGAACGTTTATGGCCCTTCCAGAAGAATACAGA
GATTATTTTATTCGAAATTTACATGATGCCTTATCAGGACATACTTCCAACAATATTTACGAGGCAGTCAAGTATTCT
GAGAGAAGCCTCACAAAGTGTATTGGAATTACTATTGAAACCAGACCAGATTACTGCATGAAGCGACATTTAAGT
GACATGTTGACCTATGGCTGCACAAGGCTGGAGATTGGGGTGCAGAGTGTTTATGAAGATGTGGCTAGAGACAC
CAACAGGGGCCACACTGTGAAGGCAGTGTGTGAGTCATTTCACCTGGCCAAAGATTCCGGTTTTAAAGTGGTGGC
CCATATGATGCCTGACCTGCCAAACGTGGGACTAGAAAGAGACATTGAACAGTTCACAGAGTTTTTTGAGAACCCT
GCTTTTCGTCCCGATGGGCTGAAACTCTATCCTACCCTGGTGATTCGTGGGACCGGGCTTTATGAGCTTTGGAAAT
CAGGAAGATATAAGAGTTACTCTCCTAGTGACCTGGTTGAATTGGTGGCTCGGATCCTAGCCCTCGTGCCTCCATG
GACTCGAGTGTACCGAGTACAGAGGGATATTCCAATGCCTTTAGTTAGCTCAGGAGTAGAGCATGGTAACCTGAG
AGAGCTGGCACTTGCAAGAATGAAAGACCTCGGAATACAGTGTCGAGATGTGAGAACCAGAGAAGTTGGAATCC
AAGAAATTCATCACAAAGTACGGCCATACCAGGTTGAATTGGTAAGGAGAGATTATGTTGCAAATGGTGGCTGGG
AAACATTCTTGTCATACGAAGACCCAGATCAAGACATTTTGATTGGCCTCCTACGATTACGCAAGTGTTCAGAAGA
AACTTTCCGTTTCGAATTGGGTGGAGGTGTCTCCATAGTACGAGAGCTGCATGTGTATGGGAGTGTGGTCCCTGT
GAGCAGCCGGGATCCTACTAAATTTCAGCATCAGGGATTTGGCATGCTGCTGATGGAGGAAGCAGAAAGAATAG
CTAGAGAAGAACATGGGTCTGGGAAAATCGCTGTGATATCAGGGGTCGGCACCAGGAATTATTATAGAAAGATC
GGCTACAGATTACAAGGCCCGTACATGGTGAAGATGCTGAAA**TAA**TGGCCACACCAGTCCACTCTTCTGCAGTATC
CTCCCTGGCAGAACACGGAGAATCAGGATTTCTTAAATACTCAACAGAGAGGCTGAGCAGAGCAAATGGGGGGC
TTCACCCTCATCCCGCAGCTGCAGAGACTGGAAACTGCCTTCAAGGCCACGGCTGGTCATCTGCTGACCACACCCC
AGATCCGCCCTCTCCTGCGTGCACCCCAAAAAATCACTTGCGTTTTTGAGGCTTAAATCATCTATCCAGTTTCTACAT
TTTGCATGAGGCCTGCAGGTGGCCTATTTTGACTCAGACGGTGAAAAAAGCAAATTAACTCATTTGGACACCATAA
CTCATGCAATAAAACTGATTGTCATTCGAGGAGCAAACTTAAGAGTAGTTTATTTATATACCCTGGGGACAGAAAG
TCAGGTTGAAACAGGAAAACCACCAGACTCTAATCTCAGCCCTTTAACGACATACGCATTGGAGCGCAAGTTAGG
AAAATGAGCTTTTGTTTTCATGGAAATCATTCTGATTACAGTGCTGATGTTTAGAAATAAATAGCAGTGTGACTGG
GAAAGAGGAATTGCAGTTGTGGGGGTGTGAGCCTGGCAGCAGCCAGCCAGCAGCCTCTCCCAGGCGGGAGTCTA
CCATCCGAGACGGCGATGACAAAGAGCTTCATTCCACATTCTTTGTTATCTCTACTTCCCACCCTCTTGGCAACTACA
GAGCAGTGTGGGCAGCCCCAAGTGTGGTCCCCAGAGAGCGTTTGGCTTTCCTGTCTGTCTATCCTGAGCGGGTGG
AGTCTCAGGTTGTGTGCCCCTAAATCAAGATTTGCTTCCACAGAAGCCATTACTTGCAATTTTTTTTTTTTTTCTGAG
AAAGTCTCGCTGTGTCACCCAGGCTGGAGTGCAGTGGCGCAATCTCACTGCATCCTCCGCCTCCCGGGTTCAAGCG
ATTCTCCCGCCTCAGCCTCCTGAGTAGCTGGGATTACAGGCACCCGCCGCTGCTAATTTTTGTATTTTTAGTAGAGA
TGGGGGTTTCACCATATTGGTCAGGCTGGTCTCGAACTCCTGACCTCAGGTGATCAACCCACCTTGGCCTCCCTAA
ATGCCGGGATTACAGGCATGAGCCACCGCTCCCAGCCTTTGATTTTTTAAGGTGGATTTTGGTTGTTATAAATGGA
GAAAGGTAAGAGTTCAAGTTCAACCCGTGTGTGAAAGCAAAACAATGGAAAACAGGATTGGCTTCTTCAAAGGCT
CCTCTTGTAGAACTGCCTCTTTGAAATTTCGAGGTAATCTACTTTGGAGACTCTGCCTGGAGAGGGTCAGTTCCTAA
GTTAAAAGCATCGCTTAACCTTGGCTCCTGTGGCATTTTACAAAGGTTTAAAGGAATTGATTCCTCTGAAAGGGCC
TGAAAATAAAAAGTCTTTAACATACAAAAAAAAAAAAAAAAAA

Protein

NCBI Reference Sequence: NP_001271154.1
LOCUS NP_001271154 428 aa

MSTHQFYRKYMCPTSMRAIRARYDPFLQTRHRIEQLKQLGHSVDKVEFIVMGGTFMALPEEYRDYFIRNLH
DALSGHTSNNIYEAVKYSERSLTKCIGITIETRPDYCMKRHLSDMLTYGCTRLEIGVQSVYEDVARDTNRGHTV
KAVCESFHLAKDSGFKVVAHMMPDLPNVGLERDIEQFTEFFENPAFRPDGLKLYPTLVIRGTGLYELWKSGRY
KSYSPSDLVELVARILALVPPWTRVYRVQRDIPMPLVSSGVEHGNLRELALARMKDLGIQCRDVRTREVGIQE
IHHKVRPYQVELVRRDYVANGGWETFLSYEDPDQDILIGLLRLRKCSEETFRFELGGGVSIVRELHVYGSVVPV
SSRDPTKFQHQGFGMLLMEEAERIAREEHGSGKIAVISGVGTRNYYRKIGYRLQGPYMVKMLK

Homo sapiens elongator acetyltransferase complex subunit 3 (ELP3), transcript variant 2, mRNA (Exon2 skipping)

mRNA

NCBI Reference Sequence: NM_001284220.2
LOCUS NM_001284220 3048bp

GGCTGTCAGCTTTCCCCGTGGTCTGAGTTTGTGGCTGCATTTTTATCTCTGGTGGCTCTGCTACGGCGGCGCAGAA
ATGAGGCAGAAGCGGAAAGGGTGAAAACCAAGACAGCTGCCAAATATGGCCTTTCTGCCCAGCCCCGCCTGGTG
GATATCATTGCTGCCGTCCCTCCTCAGTATCGCAAGGTCTTGATGCCCAAGTTAAAGGCGAAACCCATCAGAACTG
CTAGTGGGATTGCTGTCGTGGCTGTGATGTGCAAACCCCACAGATGTCCACACATCAGTTTTACAGGAAATATATG
TGTATACTGCCCTGGTGGACCTGATTCTGATTTTGAGTATTCCACCCAGTCTTACACTGGCTATGAGCCAACCTCCA
TGAGAGCTATCCGTGCCAGATATGACCCTTTCCTACAGACAAGACACCGAATAGAACAGTTAAAACAACTTGGTCA
TAGTGTGGATAAAGTGGAGTTTATTGTGATGGGTGGAACGTTTATGGCCCTTCCAGAAGAATACAGAGATTATTTT
ATTCGAAATTTACATGATGCCTTATCAGGACATACTTCCAACAATATTTACGAGGCAGTCAAGTATTCTGAGAGAA
GCCTCACAAAGTGTATTGGAATTACTATTGAAACCAGACCAGATTACTGCATGAAGCGACATTTAAGTGACATGTT
GACCTATGGCTGCACAAGGCTGGAGATTGGGGTGCAGAGTGTTTATGAAGATGTGGCTAGAGACACCAACAGGG
GCCACACTGTGAAGGCAGTGTGTGAGTCATTTCACCTGGCCAAAGATTCCGGTTTTAAAGTGGTGGCCCATATGAT
GCCTGACCTGCCAAACGTGGGACTAGAAAGAGACATTGAACAGTTCACAGAGTTTTTTGAGAACCCTGCTTTTCGT
CCCGATGGGCTGAAACTCTATCCTACCCTGGTGATTCGTGGGACCGGGCTTTATGAGCTTTGGAAATCAGGAAGAT
ATAAGAGTTACTCTCCTAGTGACCTGGTTGAATTGGTGGCTCGGATCCTAGCCCTCGTGCCTCCATGGACTCGAGT
GTACCGAGTACAGAGGGATATTCCAATGCCTTTAGTTAGCTCAGGAGTAGAGCATGGTAACCTGAGAGAGCTGGC
ACTTGCAAGAATGAAAGACCTCGGAATACAGTGTCGAGATGTGAGAACCAGAGAAGTTGGAATCCAAGAAATTC
ATCACAAAGTACGGCCATACCAGGTTGAATTGGTAAGGAGAGATTATGTTGCAAATGGTGGCTGGGAAACATTCT
TGTCATACGAAGACCCAGATCAAGACATTTTGATTGGCCTCCTACGATTACGCAAGTGTTCAGAAGAAACTTTCCG
TTTCGAATTGGGTGGAGGTGTCTCCATAGTACGAGAGCTGCATGTGTATGGGAGTGTGGTCCCTGTGAGCAGCCG
GGATCCTACTAAATTTCAGCATCAGGGATTTGGCATGCTGCTGATGGAGGAAGCAGAAAGAATAGCTAGAGAAG
AACATGGGTCTGGGAAAATCGCTGTGATATCAGGGGTCGGCACCAGGAATTATTATAGAAAGATCGGCTACAGAT
TACAAGGCCCGTACATGGTGAAGATGCTGAAATAATGGCCACACCAGTCCACTCTTCTGCAGTATCCTCCCTGGCA
GAACACGGAGAATCAGGATTTCTTAAATACTCAACAGAGAGGCTGAGCAGAGCAAATGGGGGGCTTCACCCTCAT

CCCGCAGCTGCAGAGACTGGAAACTGCCTTCAAGGCCACGGCTGGTCATCTGCTGACCACACCCCAGATCCGCCCT
CTCCTGCGTGCACCCCAAAAAATCACTTGCGTTTTTGAGGCTTAAATCATCTATCCAGTTTCTACATTTTGCATGAGG
CCTGCAGGTGGCCTATTTTGACTCAGACGGTGAAAAAAGCAAATTAACTCATTTGGACACCATAACTCATGCAATA
AAACTGATTGTCATTCGAGGAGCAAACTTAAGAGTAGTTTATTTATATACCCTGGGGACAGAAAGTCAGGTTGAAA
CAGGAAAACCACCAGACTCTAATCTCAGCCCTTTAACGACATACGCATTGGAGCGCAAGTTAGGAAAATGAGCTTT
TGTTTTCATGGAAATCATTCTGATTACAGTGCTGATGTTTAGAAATAAATAGCAGTGTGACTGGGAAAGAGGAATT
GCAGTTGTGGGGGTGTGAGCCTGGCAGCAGCCAGCCAGCAGCCTCTCCCAGGCGGGAGTCTACCATCCGAGACG
GCGATGACAAAGAGCTTCATTCCACATTCTTTGTTATCTCTACTTCCCACCCTCTTGGCAACTACAGAGCAGTGTGG
GCAGCCCCAAGTGTGGTCCCCAGAGAGCGTTTGGCTTTCCTGTCTGTCTATCCTGAGCGGGTGGAGTCTCAGGTTG
TGTGCCCCTAAATCAAGATTTGCTTCCACAGAAGCCATTACTTGCAATTTTTTTTTTTTTTTCTGAGAAAGTCTCGCTG
TGTCACCCAGGCTGGAGTGCAGTGGCGCAATCTCACTGCATCCTCCGCCTCCCGGGTTCAAGCGATTCTCCCGCCT
CAGCCTCCTGAGTAGCTGGGATTACAGGCACCCGCCGCTGCTAATTTTTGTATTTTTAGTAGAGATGGGGGTTTCA
CCATATTGGTCAGGCTGGTCTCGAACTCCTGACCTCAGGTGATCAACCCACCTTGGCCTCCCTAAATGCCGGGATT
ACAGGCATGAGCCACCGCTCCCAGCCTTTGATTTTTTAAGGTGGATTTTGGTTGTTATAAATGGAGAAAGGTA
AGAGTTCAAGTTCAACCCGTGTGTGAAAGCAAAACAATGGAAAACAGGATTGGCTTCTTCAAAGGCTCCT
CTTGTAGAACTGCCTCTTTGAAATTTCGAGGTAATCTACTTTGGAGACTCTGCCTGGAGAGGGTCAGTTC
CTAAGTTAAAAGCATCGCTTAACCTTGGCTCCTGTGGCATTTTACAAAGGTTTAAAGGAATTGATTCCTC
TGAAAGGGCCTGAAAATAAAAAGTCTTTAACATACAAA

Protein

NCBI Reference Sequence: NP_001271149.1
LOCUS   NP_001271149   475 aa

MPKLKAKPIRTASGIAVVAVMCKPHRCPHISFTGNICVYCPGGPDSDFEYSTQSYTGYEPTSMRAIRARYDPF
LQTRHRIEQLKQLGHSVDKVEFIVMGGTFMALPEEYRDYFIRNLHDALSGHTSNNIYEAVKYSERSLTKCIGITI
ETRPDYCMKRHLSDMLTYGCTRLEIGVQSVYEDVARDTNRGHTVKAVCESFHLAKDSGFKVVAHMMPDLP
NVGLERDIEQFTEFFENPAFRPDGLKLYPTLVIRGTGLYELWKSGRYKSYSPSDLVELVARILALVPPWTRVYRV
QRDIPMPLVSSGVEHGNLRELALARMKDLGIQCRDVRTREVGIQEIHHKVRPYQVELVRRDYVANGGWETF
LSYEDPDQDILIGLLRLRKCSEETFRFELGGGVSIVRELHVYGSVVPVSSRDPTKFQHQGFGMLLMEEAERIAR
EEHGSGKIAVISGVGTRNYYRKIGYRLQGPYMVKMLK

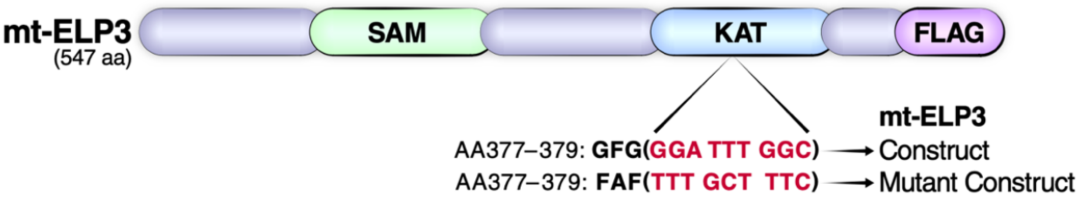

Figure S4. Schematic view of the domain structure of mt-ELP3 construct. The position of the change introduced in mt-ELP3 to generate mt-ELP3 mutant is highlighted.

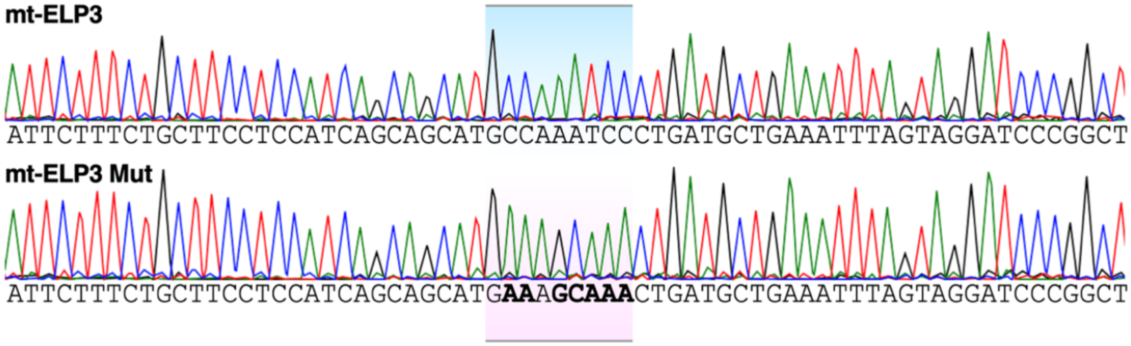

Figure S5. Part of the sequencing results**.** The mutation: amino acid (AA) 377-379: GFG(GGATTTGGC)➔FAF(TTTGCTTTC) is highlighted.

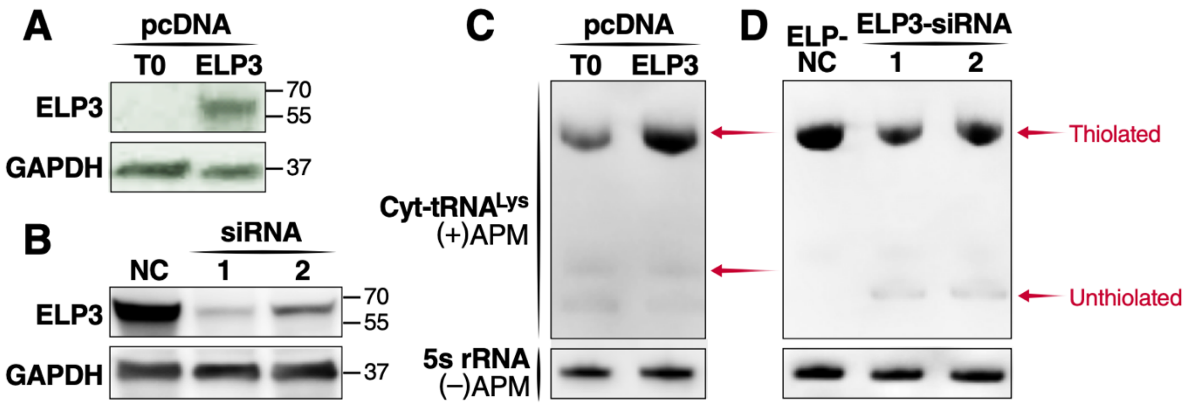

Figure S6. Overexpression or transient silencing of canonical ELP3 in HEK293T cells affects the 2 thiolation modification status of cyt-tRNALys molecules.

(A and B) Western blot analysis of canonical ELP3 expression in HEK293T cells transfected with empty plasmid (pcDNA4 T0) and pcDNA ELP3 (A) and in ELP3 siRNA 1, ELP3 siRNA 2- and Negative Control (NC) siRNA-transfected HEK293T cells (B). The membranes were also probed with an antibody against GAPDH, which was used as a loading control.

(C and D) APM-northern analysis of the 2-thiolation status of mt-tRNALys molecules isolated from HEK293T transfected with pcDNA4 T0 and pcDNA ELP3 (C) and from ELP3 siRNA 1, ELP3 siRNA 2- and Negative Control (NC) siRNA-transfected HEK293T cells (D). The same amount of total RNA (5 μg) was run in a denaturing polyacrylamide-urea gel in the presence (+) or absence (-) of APM. The thiolated tRNAs were detected as retarded bands in the presence of APM. The APM (−) membrane was probed with 5S rRNA as a loading control.

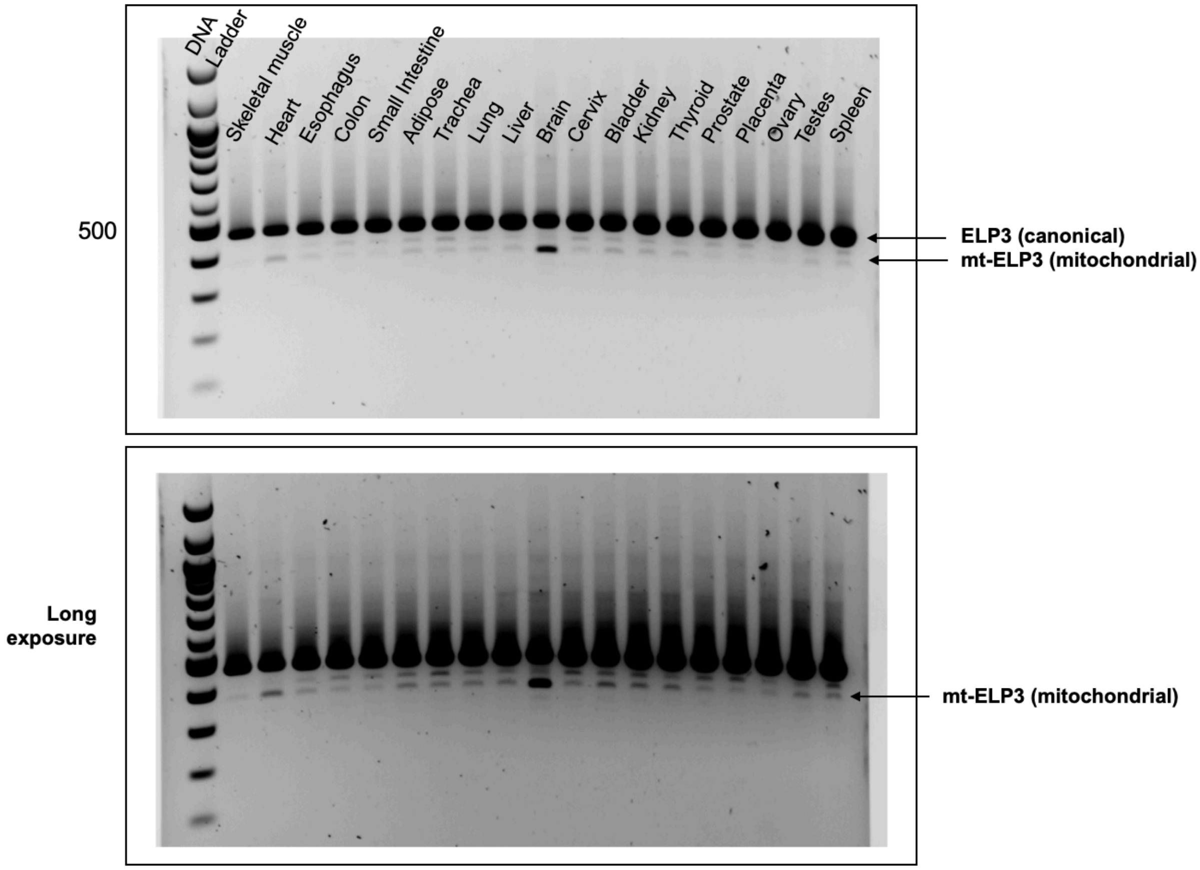

Figure S7. mt-ELP3 is abundant in brain tissue.

PCR analysis of mt-ELP3 expression in different human tissues using exon1-6 spanning primers (table S1).

Table S1. ELP3 primes and digoxigenin (DIG)-labeled oligodeoxynucleotide probes

Gene name	Oligonucleotide name	Sequence (5'→3')	Assay
ELP3	ELP3-Fw	TGGCTGCATTTTTATCTCTGG	**qRT-PCR**
	ELP3-Rv	CAGGCACGGATAGCTCTCAT	
mt-tRNA^{Lys}	mt-tRNALys	(Dig) TGGTCACTGTAAAGAGGTGTTGGT	**Northern blot analysis**
mt-tRNA^{Val}	mt-tRNAVal	(Dig) GAAATCTCCTAAGTGTAAGTTGGGTGCTTTG	**Northern blot analysis**
mt-tRNA^{Glu}	mt-tRNAGlu	(Dig) GACTACAACCACGACCAATGATATGAAAAAC	**Northern blot analysis**
mt-tRNA^{Leu}	mt-tRNALeu	(Dig) GGAATTGAACCTCTGACTGTAAAGTTTTAAG	**Northern blot analysis**
5 rRNA	5 rRNA	(Dig) GGGTGGTATGGCCGTAGAC	**Northern blot analysis**

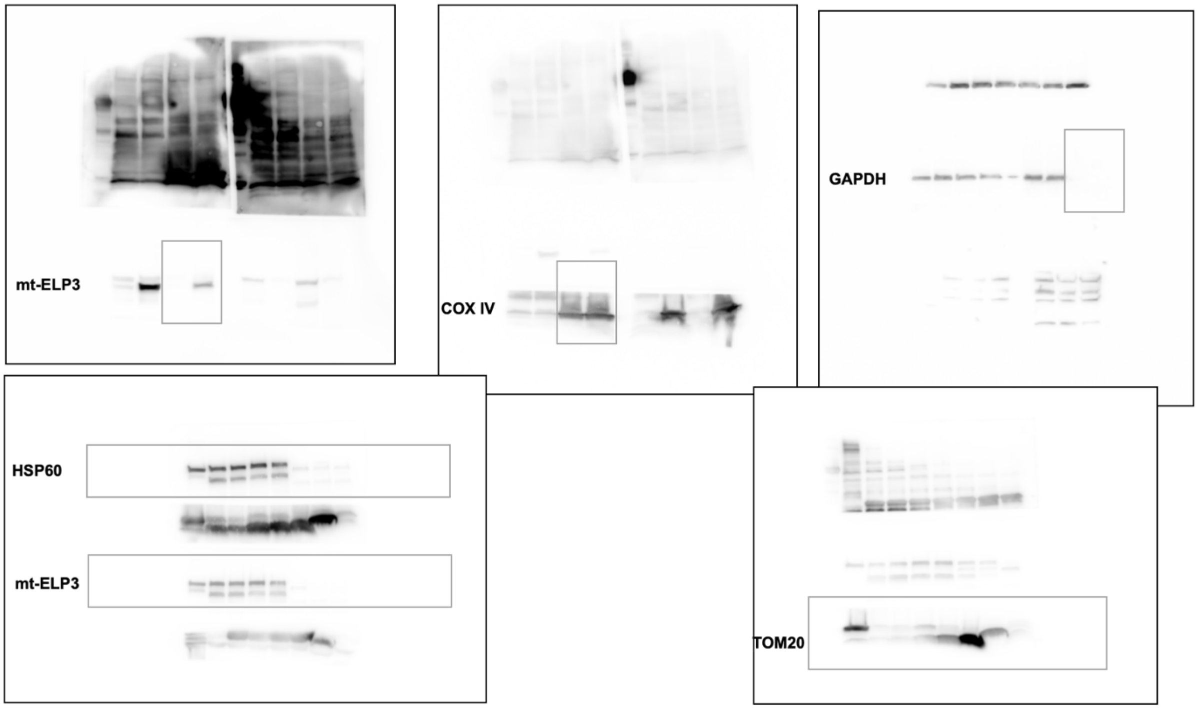

Figure S8 (1): Full-length Western blots of Figures 1A and 2B. Membranes were cut horizontally before probing with antibodies. The blots were obtained using the Azure Biosystems c600 imager and the edge of the membranes is not always visible. Many members were developed simultaneously; thereby, the area cropped and shown in Figure 1 is indicated by a rectangle.

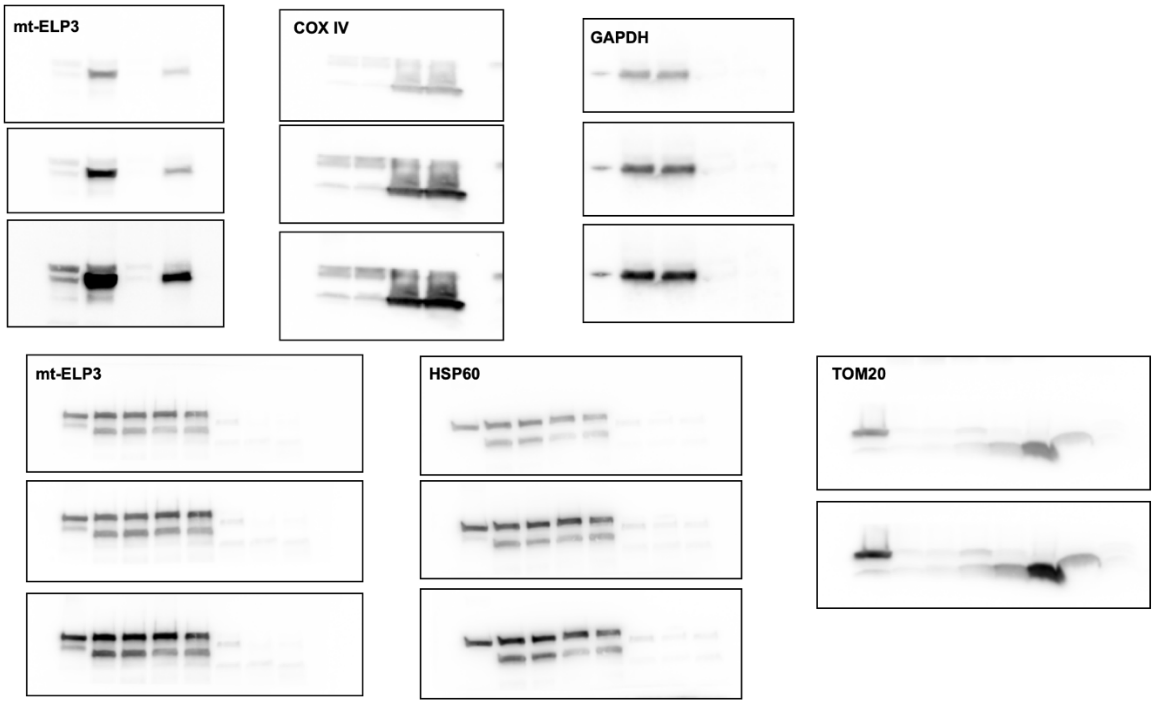

Figure S8 (2): Multiple exposure images

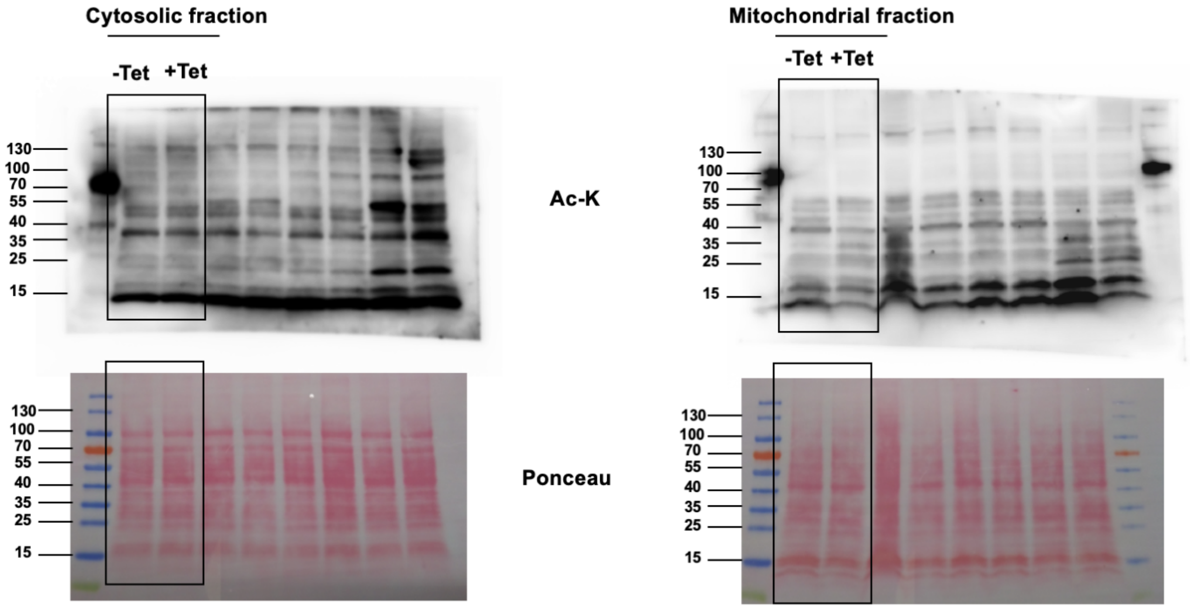

Figure S9: Full-length Western blots of Figure 2. The full-length membrane was stained with Ponceau before incubation with blocking and pan-acetyllysine antibody. The area cropped and shown in Figure 2 is indicated by a rectangle.

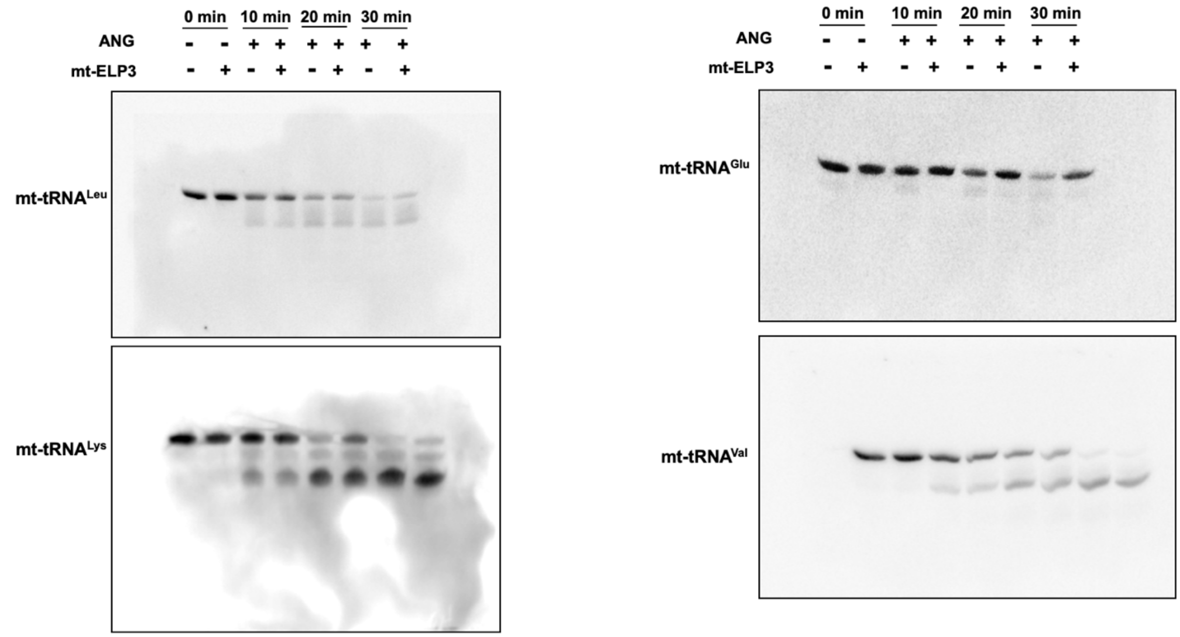

Figure S10 (1): Full-length Northern blots of Figure 3A. The full-length membrane was incubated with the indicated probe. The blots were obtained using the Azure Biosystems c600 imager and the edge of the membranes is not always visible.

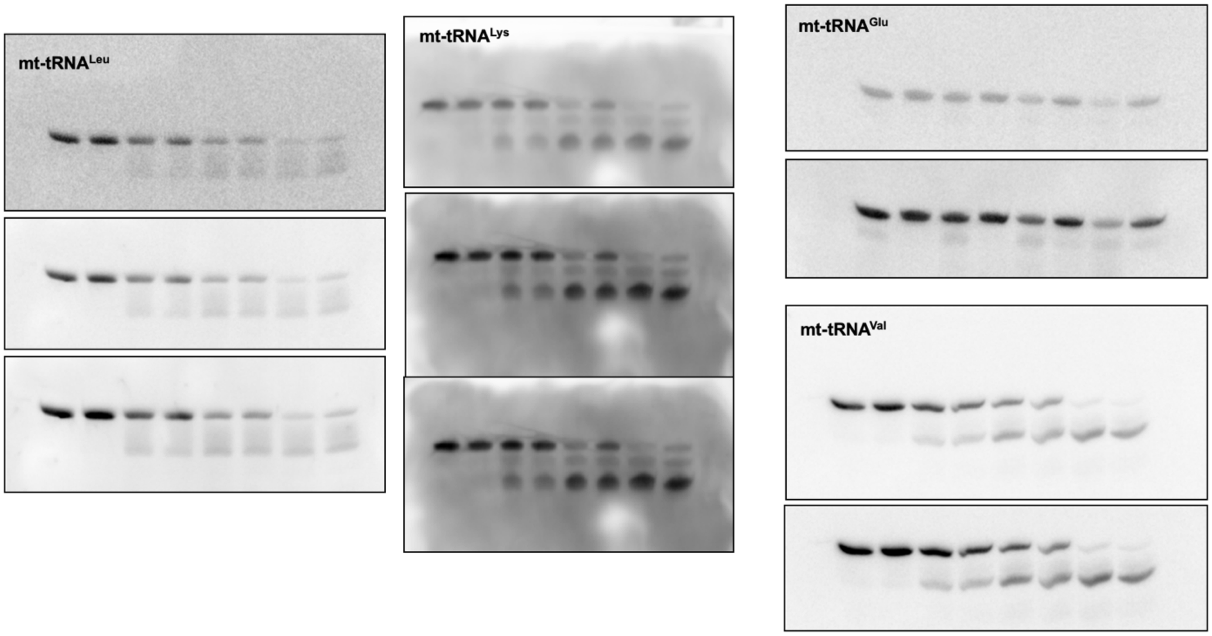

Figure S10 (2): Multiple exposure images

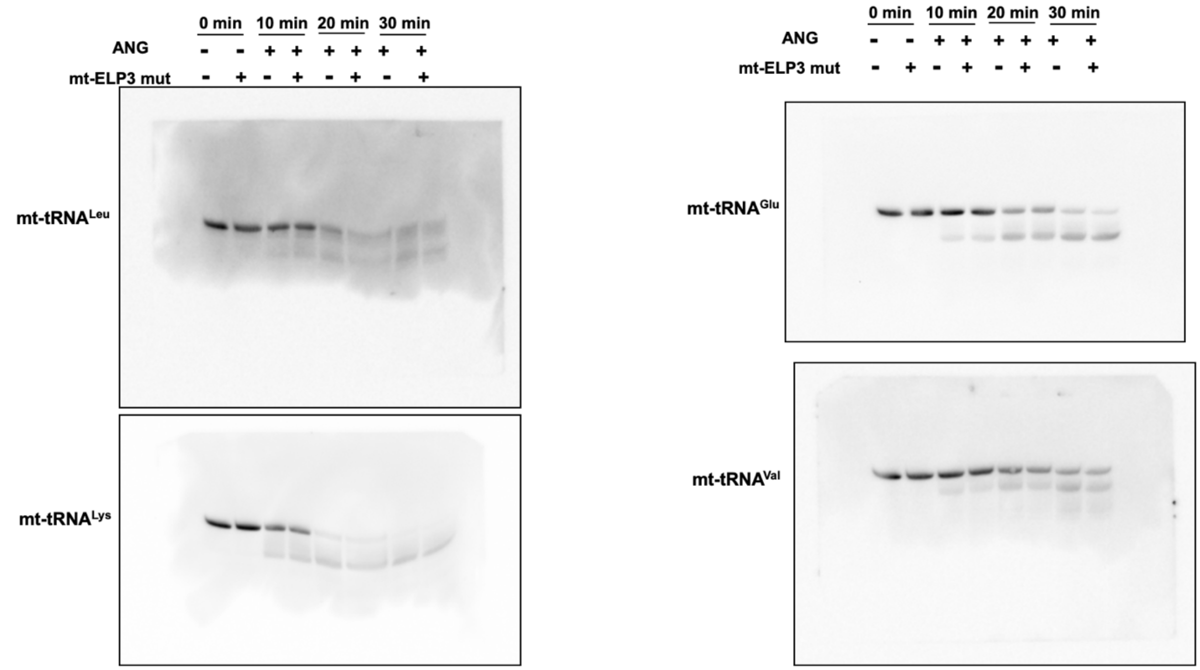

Figure S11 (1): Full-length Northern blots of Figure 3B. The full-length membrane was incubated with the indicated probe. The blots were obtained using the Azure Biosystems c600 imager and the edge of the membranes is not always visible.

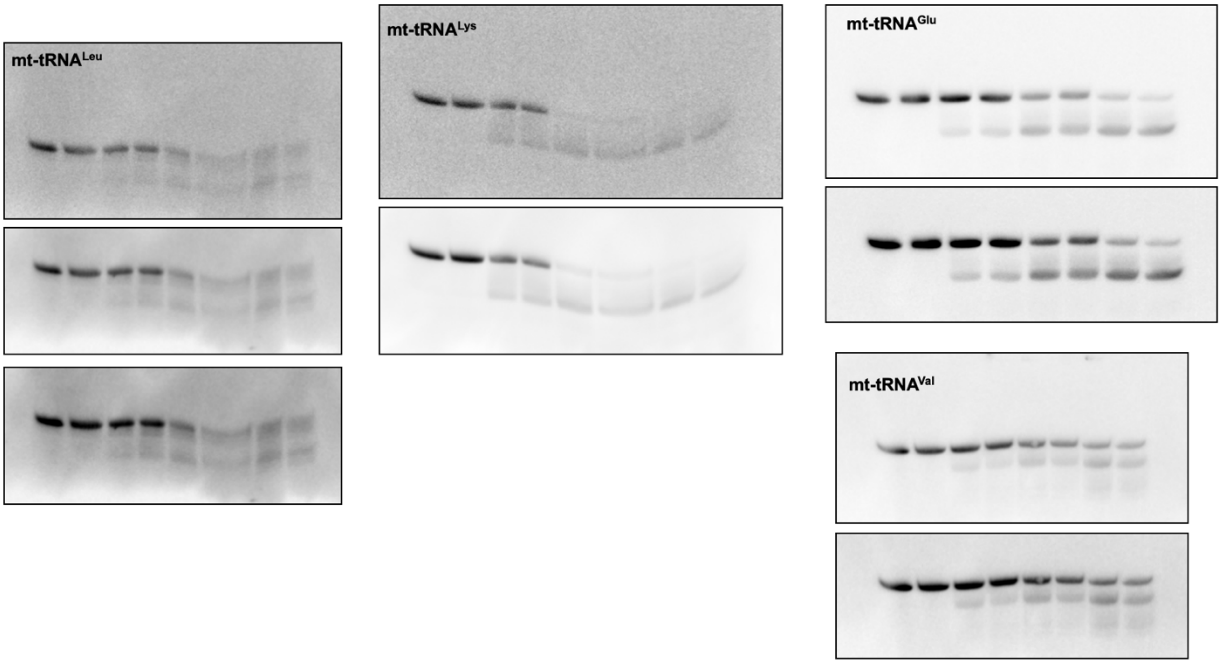

Figure S11 (2): Multiple exposure images

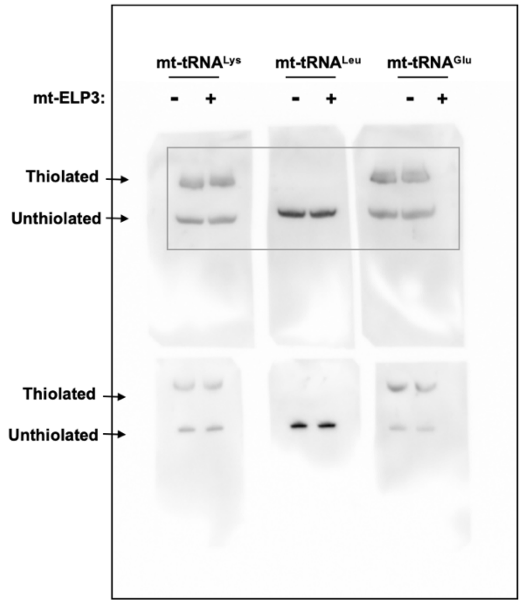

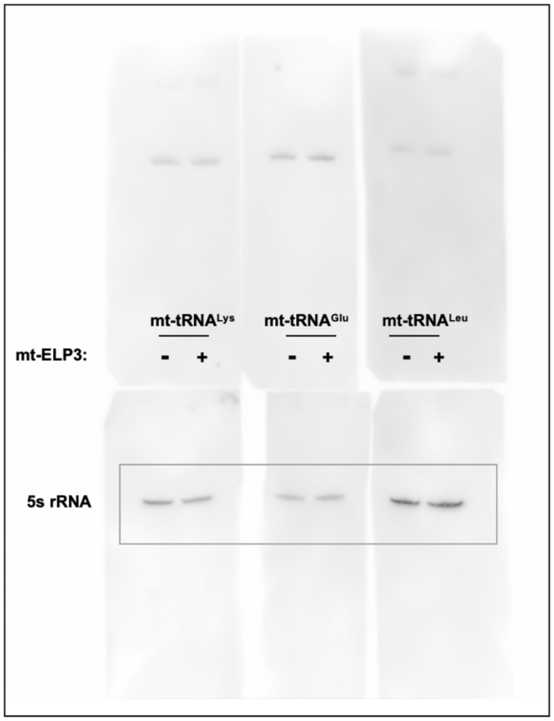

Figure S12 (1): Full-length Northern blots of Figure 3C. Membranes were cut vertically before probing with the indicated probe. The blots were obtained using the Azure Biosystems c600 imager. Many members were developed simultaneously; thereby, the area cropped and shown in Figure 3C is indicated by a rectangle.

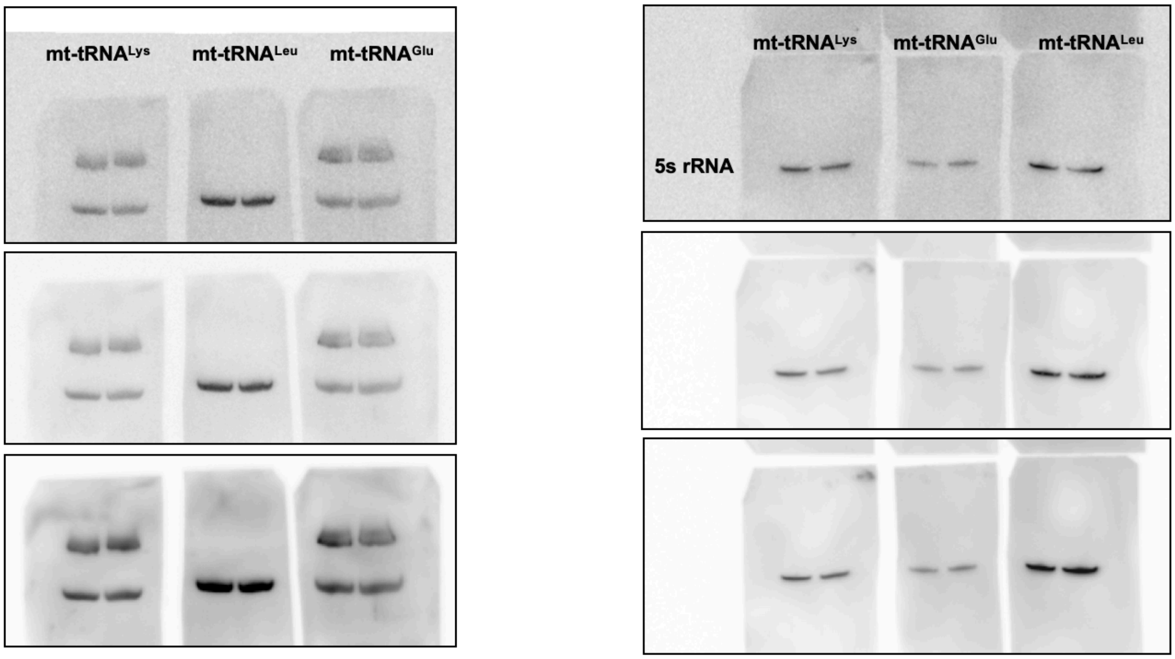

Figure S12 (2): Multiple exposure images

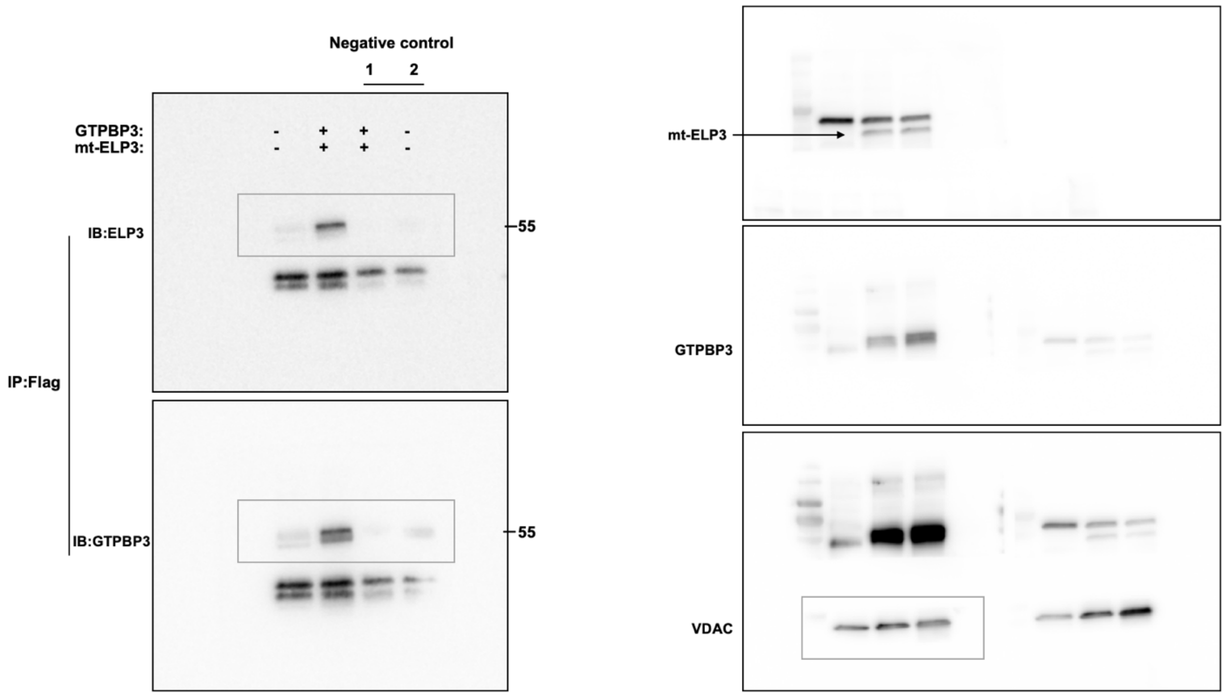

Figure S13 (1): Full-length Western blots of Figure 3D. Membranes were cut horizontally before probing with antibodies. The blots were obtained using the Azure Biosystems c600 imager and the edge of the membranes is not always visible. Many members were developed simultaneously; thereby, the area cropped and shown in Figure 3D is indicated by a rectangle.

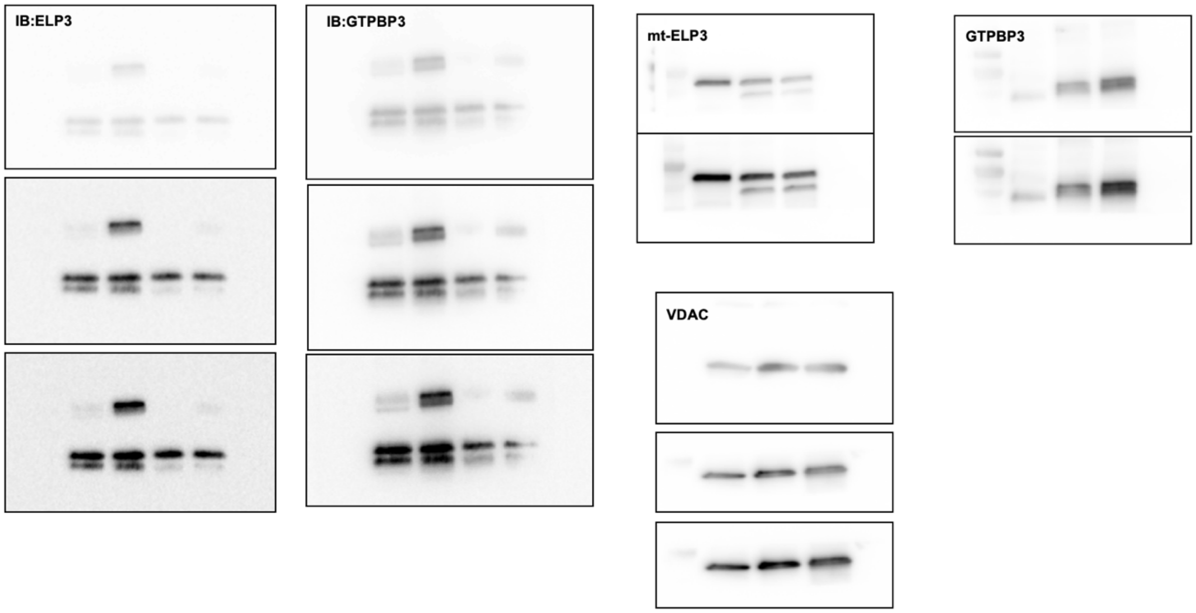

Figure S13 (2): Multiple exposure images

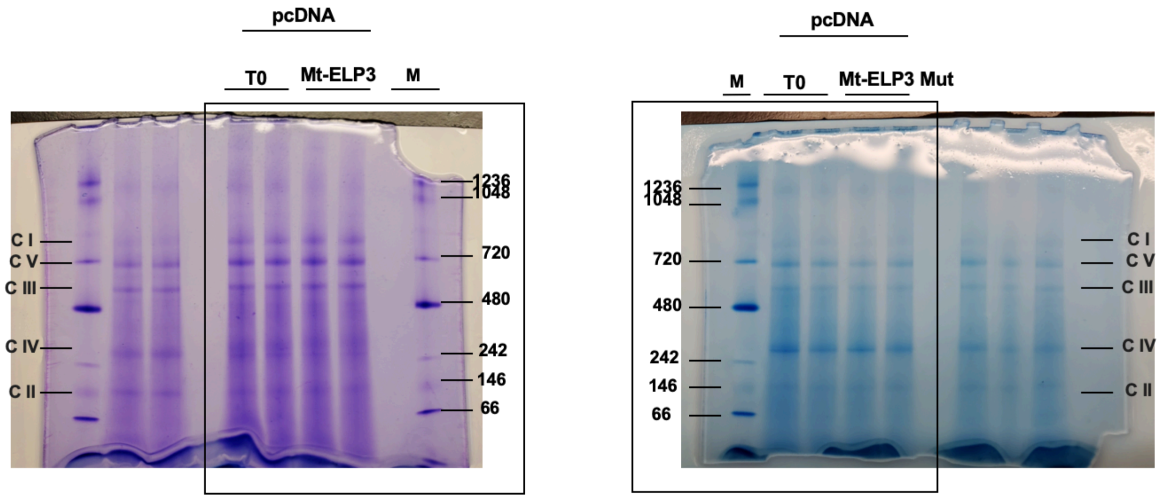

Figure S14: Full-length of representative BN-PAGE gel stained with Coomassie Brilliant Blue of figures 5A and 5C. The area cropped is indicated by a rectangle.

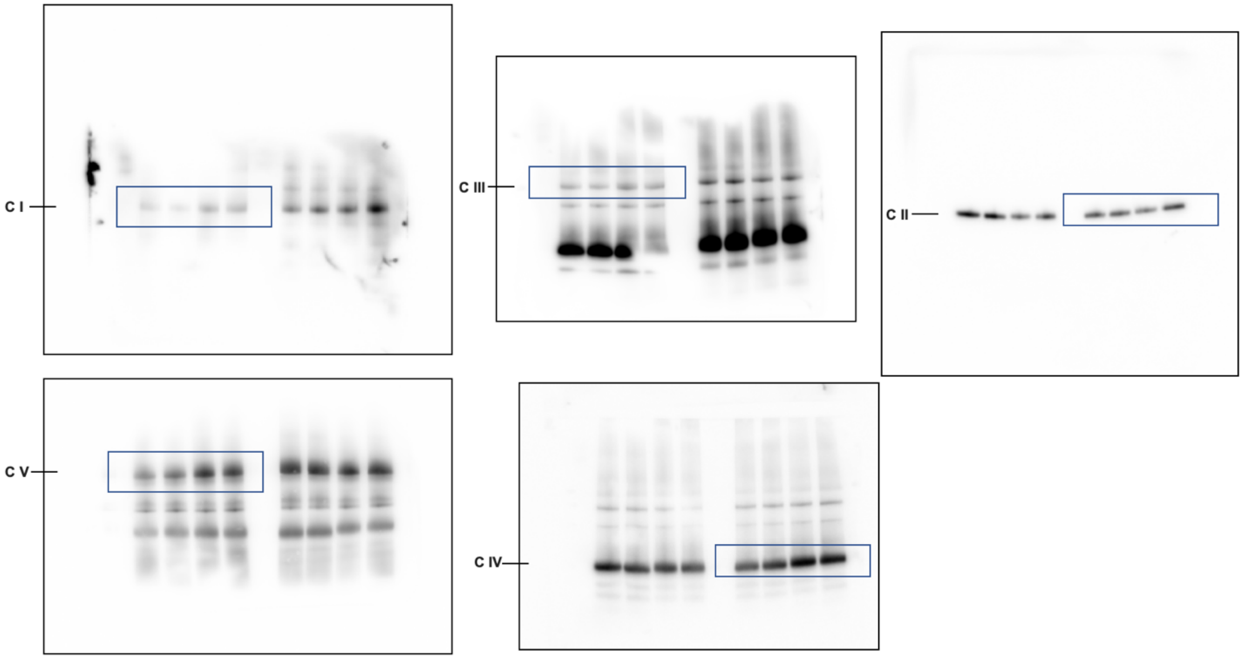

Figure S15 (1): Full-length Western blots of Figure 5B. The full-length membrane was incubated with the indicated antibodies. The blots were obtained using the Azure Biosystems c600 imager and the edge of the membranes is not always visible. The area cropped and shown in Figure 5B is indicated by a rectangle.

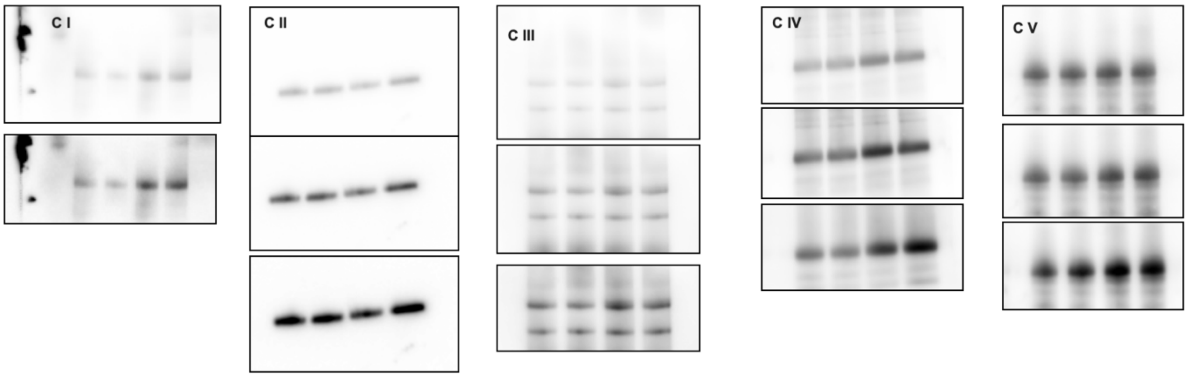

Figure S15 (2): Multiple exposure images

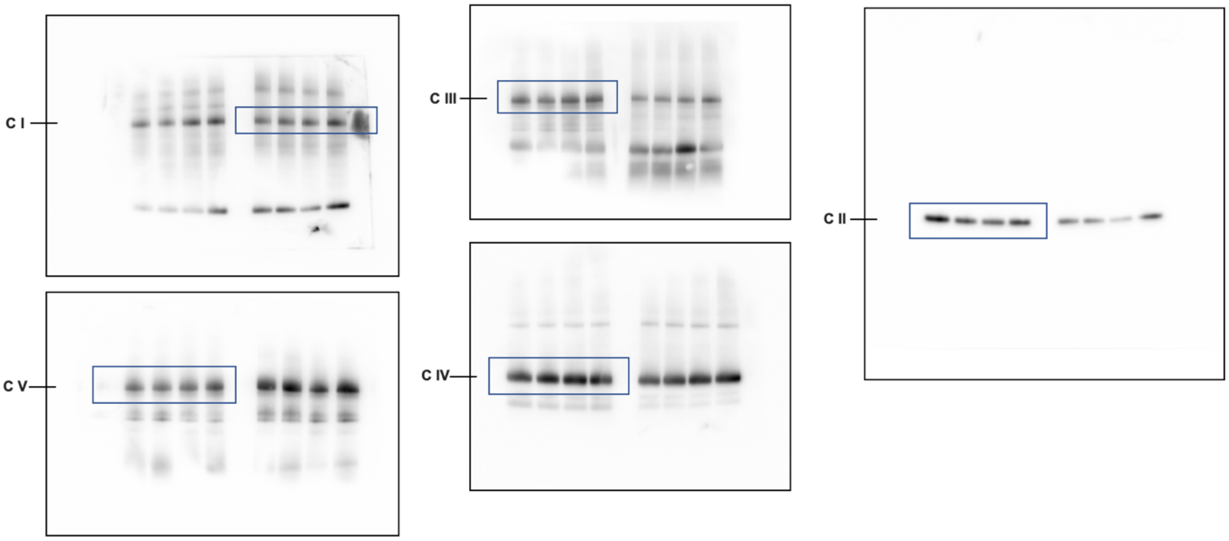

Figure S16 (1): Full-length Western blots of Figure 5D. The full-length membrane was incubated with the indicated antibodies. The blots were obtained using the Azure Biosystems c600 imager and the edge of the membranes is not always visible. The area cropped and shown in Figure 5D is indicated by a rectangle.

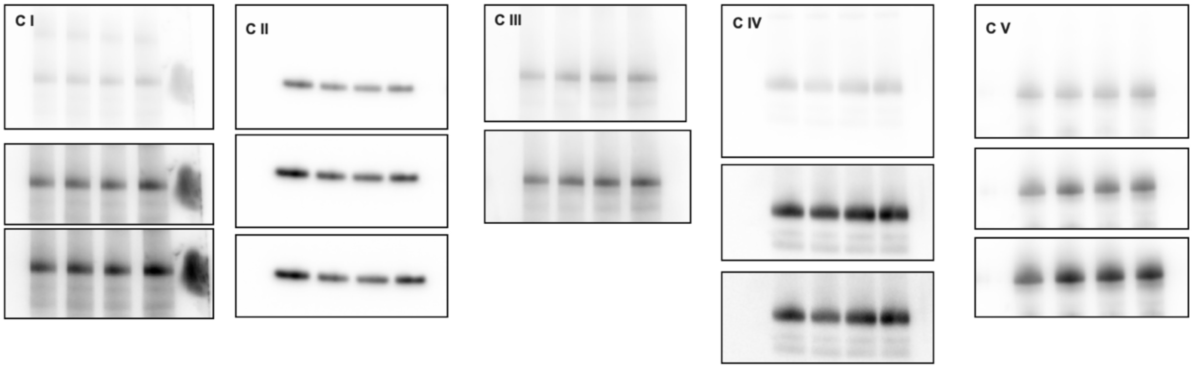

Figure S16 (2): Multiple exposure images

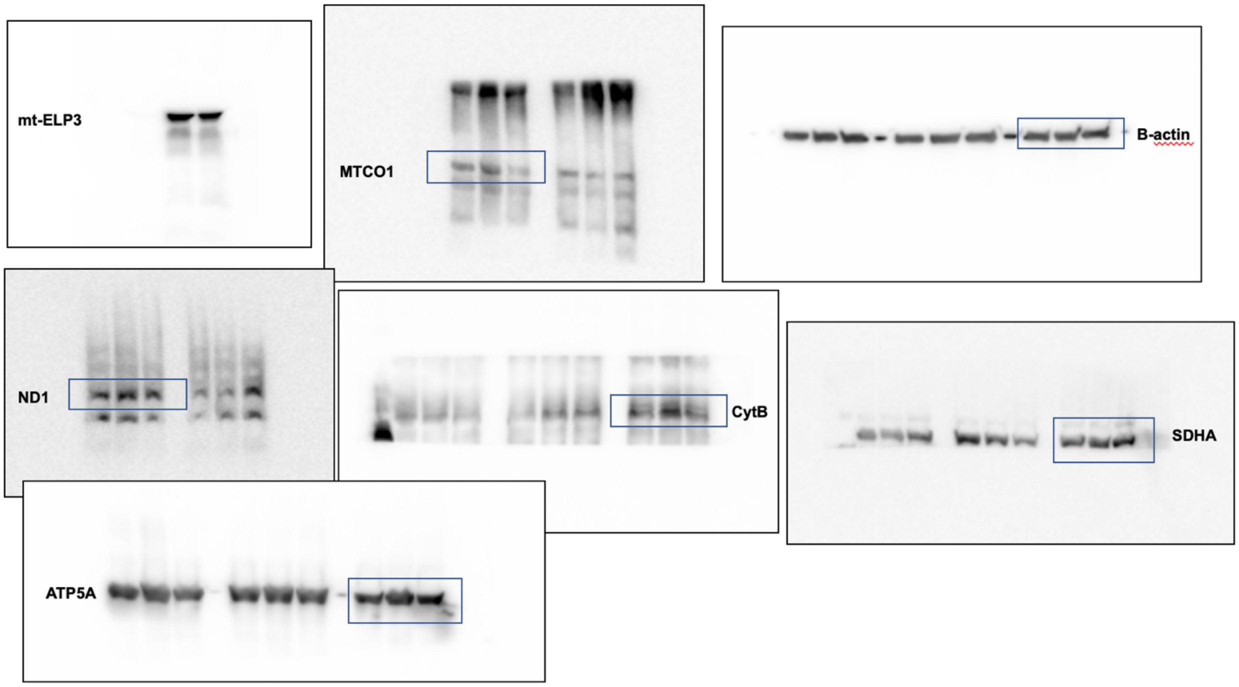

Figure S17 (1): Full-length Western blots of Figure 5E. Membranes were cut horizontally before probing with antibodies. The blots were obtained using the Azure Biosystems c600 imager and the edge of the membranes is not always visible. The area cropped and shown in Figure 5E is indicated by a rectangle.

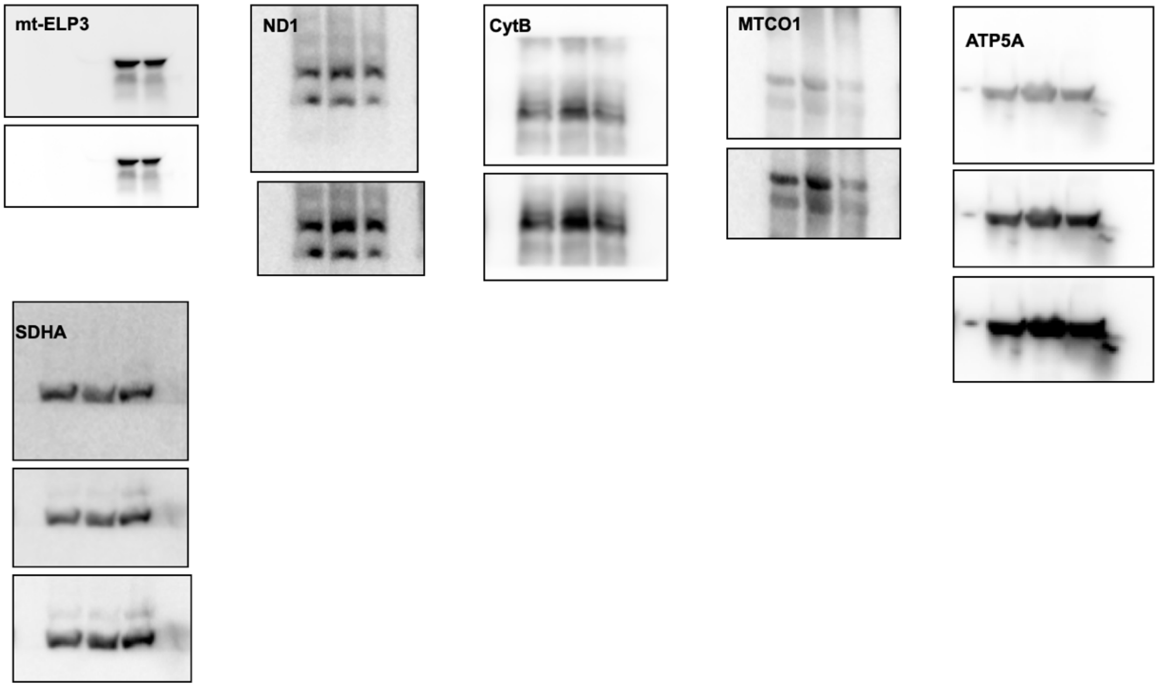

Figure S17 (2): Multiple exposure images

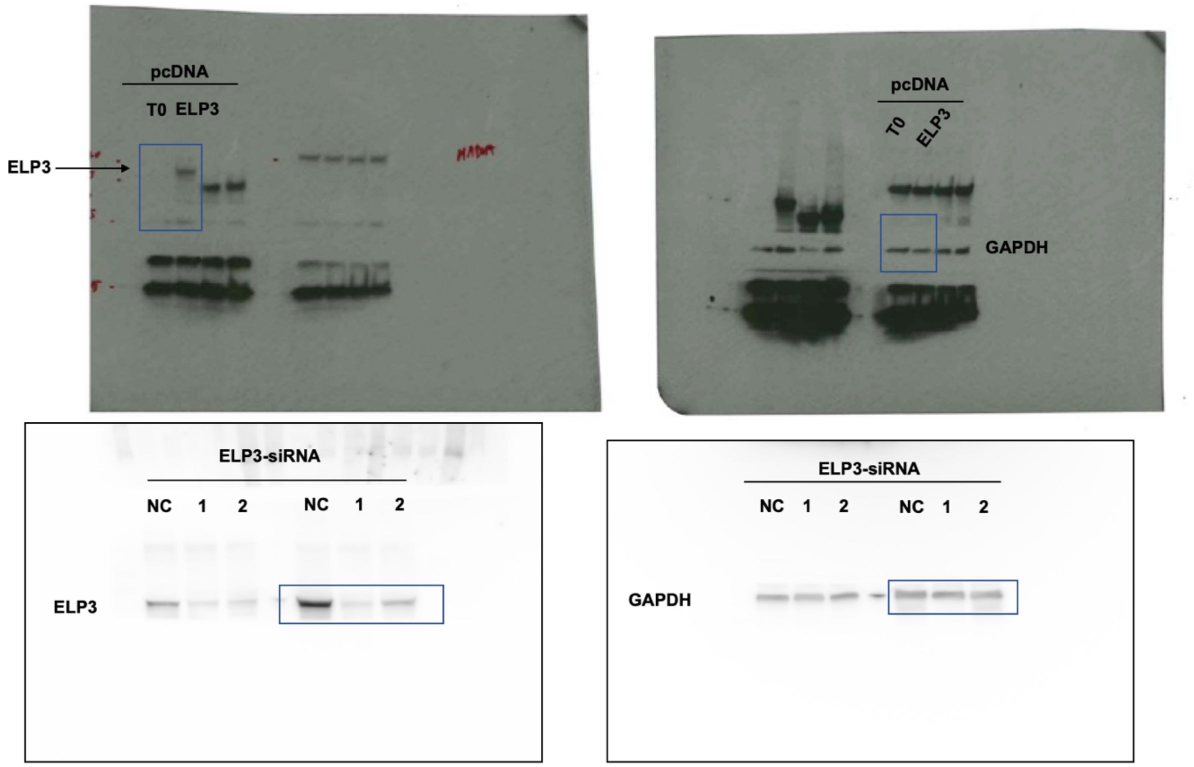

Figure S18 (1): Full-length Western blots of Figure S6A and B. Membranes were cut horizontally before probing with antibodies. The upper part was obtained using x-ray films and the lower one using the Azure Biosystems c600 imager. The edge of the membranes is not always visible. Many members were developed simultaneously; thereby, the area cropped and shown in Figure S6A and B is indicated by a rectangle.

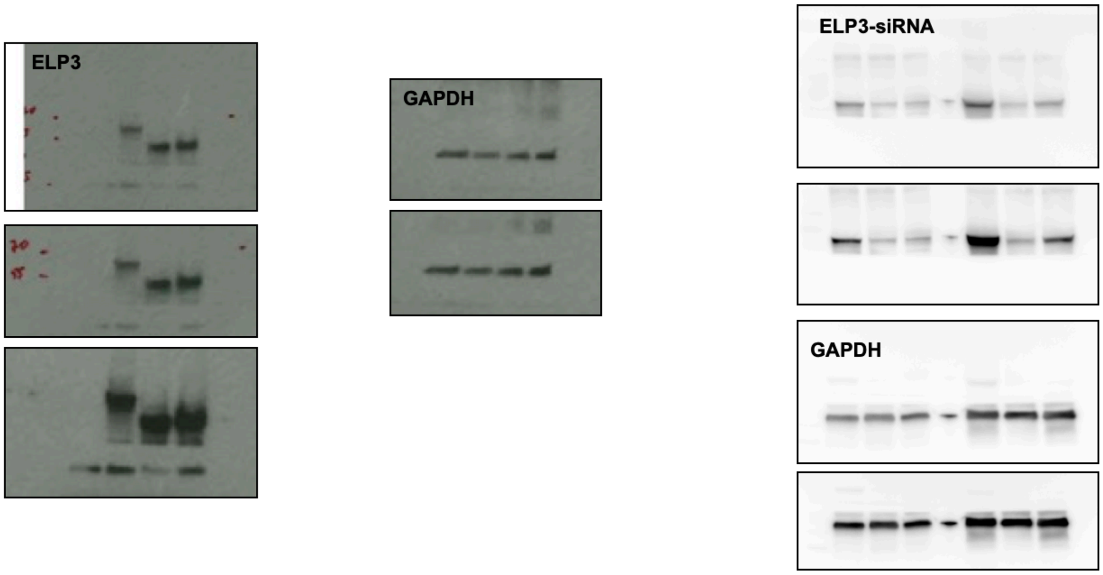

Figure S18 (2): Multiple exposure images

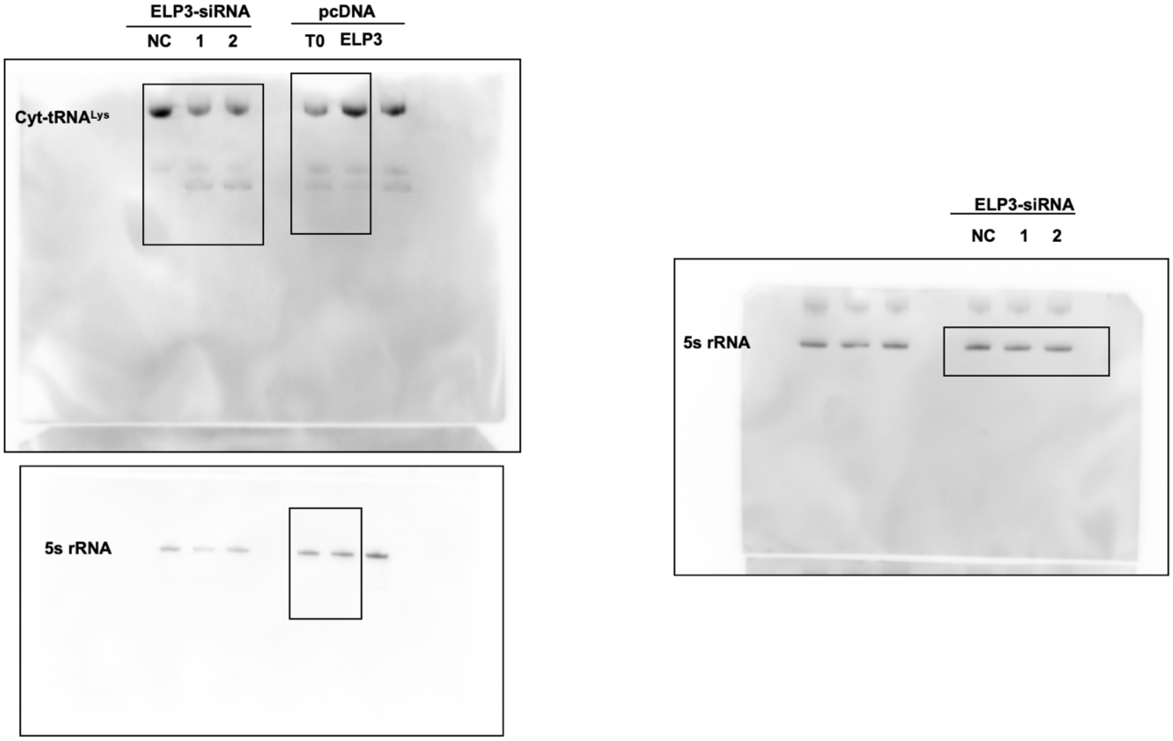

Figure S19 (1): Full-length Western blots of Figure S6C and D. The full-length membrane was incubated with the indicated probe. The blots were obtained using the Azure Biosystems c600 imager and the edge of the membranes is not always visible. The area cropped and shown in Figure S6C and D is indicated by a rectangle.

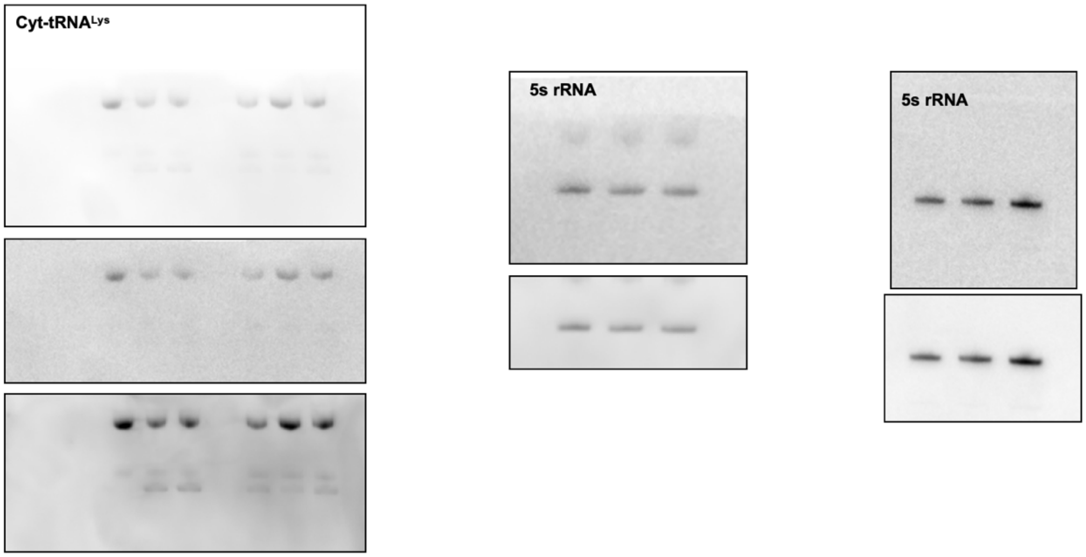

Figure S19 (2): Multiple exposure images
